# Effect of Exogenous Progesterone or Flunixin Meglumine After AI on Serum Progesterone Concentration and Pregnancy per AI in Lactating Dairy Cows

**DOI:** 10.21451/1984-3143-AR2017-0014

**Published:** 2018-08-16

**Authors:** Saber Barkhori-Mehni, Hamed Karami-Shabankareh, Reza Masoumi, Mehdi Kazemi- Bonchenari, Adel Pezeshki, Arya Badiei, Essa Dirandeh, Marcos G. Colazo

**Affiliations:** 1 Department of Health and Food Safety, Faculty of Veterinary Medicine, Ferdowsi University of Mashhad, Mashhad, Iran.; 2 Department of Animal Science, Faculty of Agriculture, University of Razi, Kermanshah, Iran.; 3 Department of Animal Science, Faculty of Agriculture, University of Zanjan, Zanjan, Iran.; 4 Department of Animal Science, Faculty of Agriculture and Natural Resources, Arak University, Arak, Iran.; 5 Department of Animal Science, Oklahoma State University, Stillwater, United States of America.; 6 Department of Clinical Sciences, Veterinary Faculty, Islamic Azad University, Karaj, Alborz, Iran.; 7 Department of Animal Science, Sari Agricultural Sciences and Natural Resources University, Sari, Iran.; 8 Livestock Research Section, Alberta Agriculture and Forestry, Edmonton, AB, T6H 5T6, Canada.

**Keywords:** embryo loss, cattle, progesterone, reproductive performance

## Abstract

The objective of this study was to determine the effect of post AI administration of exogenous progesterone (P_4_) or a prostaglandin F2α (PGF2α) synthesis inhibitor agent on serum P_4_ concentrations and pregnancy per AI (P/AI) in lactating dairy cows. Eighty lactating cows were randomly allocated to one of four treatment groups: 1) CON (control), received 5 mL of saline solution on d 6 and 14 post AI; 2) IP4 (injection of P _4_), received 125 mg of P_4_ im on d 6 and 14 post AI; 3) CIDR, received a controlled internal drug release insert containing 1.38g of P_4_ from d 6 to 20 post AI; and 4) FM (Flunixin Meglumine), received 0.625 g of Flunixin Meglumine, a nonsteroidal anti-inflammatory drug, twice daily on d 15 and 19 post AI. Blood samples were taken on d 0, 6, 14, 17 and 20 post AI to determine P_4_ concentrations. Transrectal palpation was performed between 40 and 45 d post AI to determine pregnancy status. All treatment groups (i.e. IP4, CIDR and FM) resulted in greater serum P_4_ concentration on d 17 and 20 post AI compared to CON (P < 0.05). Cows given a CIDR insert had greater concentrations of P_ 4_ on d 17 and 20 than IP4 and FM cows (P < 0.05). However, no significant difference was found between IP4 and FM groups for serum P_4_ concentrations. The P/AI was greater (P < 0.05) in CIDR-treated cows (55%, 11/20) than CON (25%, 5/20), and intermediate in IP4 (40%, 8/20) and FM (35%, 7/20) cows. In summary, treatment with exogenous P_4_ (i.e. CIDR and IP4) or FM increased serum P_4_ concentrations in lactating dairy cows. However, results suggest that only CIDR administration would improve P/AI.

## Introduction

Early embryonic development and establishment of pregnancy in cattle depends upon progesterone (P_4_) secretion by the corpus luteum (CL; Santos *et al*., 2004 ; Leroy *et al*., 2008 ; Motavalli *et al*., 2017 ). Reduced pregnancy per AI (P/AI) in dairy cattle has been associated with low circulating P _4_ concentrations or a delay in the rise of P_4_ during the early post ovulatory phase ( Mann and Lamming, 1999 ; Masoumi *et al*., 2012 ; Masoumi *et al*., 2017 ). Moreover, high producing dairy cows have increased dry matter intake ( Holter *et al*., 1997 ; Badiei *et al*., 2014 ), hepatic blood flow ( Sangsritavong *et al*., 2002 ), and steroid hormone metabolism resulting in inadequate concentrations of circulating P _4_. Therefore, various studies have evaluated the effect of supplementation with exogenous P_4_ during metestrus or early diestrus on fertility of dairy cows, but results have been inconsistent ( Larson *et al*., 2007 ; Arndt *et al*., 2009 ; Friedman *et al*., 2012 ; Colazo *et al*., 2013 ). In this regard, the efficacy of P_4_ supplementation post-AI on pregnancy success was recently evaluated in a meta-analysis including 84 treatments involving data from 19,040 cows ( Yan et *al*., 2016 ). Results showed that P_4_ supplementation from d 3 to 7 post-AI was beneficial but supplementation either earlier or later than this period reduced or did not affect P/AI. However, in a recent study, Garcia-Ispierto and López-Gatius (2017) compared pregnancy risk in high-producing lactating dairy cows administered exogenous P_ 4_ during metaestrus (3 to 5 d post AI) or during the time of pregnancy recognition (15 to 17 d post-AI) with that in untreated control cows. Cows treated with P_4_ during metaestrus or during the time of pregnancy recognition were 1.71 and 1.4 times, respectively, most likely to become pregnant than untreated control cows.

In cattle, the regression of the CL (luteolysis) is initiated by the release of uterine prostaglandin F_2_α (PGF_2_α) at the late luteal stage ( McCracken *et al*., 1999 ; Masoumi *et al*., 2011 ). Hence, other studies have attempted to extend the life-span of the CL with the administration of agents that inhibit the synthesis or release of PGF_2_α to enhance pregnancy in cattle ( Elli *et al*., 2001 ; Geary *et al*., 2010 ). In this regard, greater P/AI was obtained in Italian Friesian cattle treated with ibuprofen lysinate, a Cyclooxygenase 1 and 2 (COX-1 and COX-2) inhibitor on d 17 after AI ( Elli *et al*., 2001 ). Flunixin Meglumine (FM), a strong non-steroid anti-inflammatory drug (NSAID), also prevents the synthesis of COXs and conversion of arachidonic acid to PGF_2_α ( Purcell *et al*., 2005 ; Geary *et al*., 2010 ). Although, Anderson *et al.* (1986) reported that FM could potentially extend the life-span of the CL in lactating dairy cows, there is a lack of publications about the effect of FM on serum concentration of P_4_ and P/AI.

The primary objective of this study was to evaluate the effect of post AI administration of exogenous P_4_ or a PGF_2_α synthesis inhibitor agent on serum P_4_ concentrations in lactating dairy cows. A secondary objective was to determine whether post AI administration of exogenous P_4_ or a PGF_2_α synthesis inhibitor agent would improve P/AI. Our hypothesis was that post AI administration of exogenous P_ 4_ or a PGF_2_α synthesis inhibitor agent would increase serum P_ 4_ concentrations and the proportion of cows becoming pregnant after AI.

## Material and methods

This study was carried out on a commercial dairy farm located near Kermanshah, Iran. All animal experimental procedures were reviewed and approved by the Iranian Ministry of Agriculture (experimental permission No. 3548). Eighty multiparous Holstein dairy cows (Mean ± SEM; body weight: 652 ± 22 Kg; days in milk: 83 ± 15, milk production 29.8 ± 1.6 L/d; parity: 3.34 ± 0.58, body condition score: 2.84 ± 0.26) were used in the present study. Cows were fed a TMR (Total Mixed Ration) formulated for a lactating dairy cow of 650 kg body weight, producing 35 kg of 3.5% fat milk per day, according to NRC (2001). The TMR was offered thrice daily and cows had free access to water. Estrus detection was performed by two technicians every 3 h for at least 20 min during each observation. Estrus was confirmed by transrectal palpation before AI as previously described ( López-Gatius and Camón-Urgel, 1991 ; Masoumi *et al*., 2018 ). Cows confirmed in estrus were inseminated, according to the am/pm rule, with semen from sires available commercially. All cows were inseminated by the same technician. Pregnancy status was determined by transrectal palpation performed between 40 and 45 d post AI.

The day of AI was considered as day 0 of the study and cows (n = 20/group) were randomly allocated to one of four treatment groups (i.e. CON, IP4, CIDR and FM). Cows in the CON group received 5 mL of saline solution on d 6 and 14 post AI. Cows in IP4 group received 125 mg of P_4_ (25 mg/mL P_4_, Aburaihan Pharmaceutical Co., Tehran, Iran) on d 6 and 14 post AI. Cows in the CIDR group received a controlled internal drug release insert containing 1.38g of P_4_ (EAZI-BREED CIDR, Zoetis Inc., Dublin, Ireland) from d 6 to 20 post AI. Cows in the FM group received 0.625 g of Flunixin Meglumine (50 mg/mL, Banamine, Merck Animal Health, Darmstadt, Germany) twice daily on d 15 and 19 post AI. All treatments were carried out at approximately 0007 h.

Blood samples (~10 mL) were collected from coccygeal vein into evacuated tubes (Vacutainer®; Becton Dickinson and Company, New Jersey, USA) on d 0, 6, 14, 17 and 20 post AI in all cows approximately at 1100 h. Blood samples were left undisturbed for approximately 2 h at room temperature to allow clot formation and then centrifuged at 2000 × g for 10 min, serum harvested and frozen at -20°C until assayed for P_4_. The serum P_4_ concentration was determined using a commercial ELISA kit (DRG, Marburg, Germany; EIA-1292) as described previously ( Heidari *et al*., 2017 ; Masoumi *et al*., 2017 ). The sensitivity of assay was 0.2 ng/mL and the inter- and intra-assay coefficients of variation for samples were 10.2 and 8.3%, respectively. The inter- and intra-assay coefficients of variation for control values were 2.2 and 3.0%, respectively.

**Figure 1:**
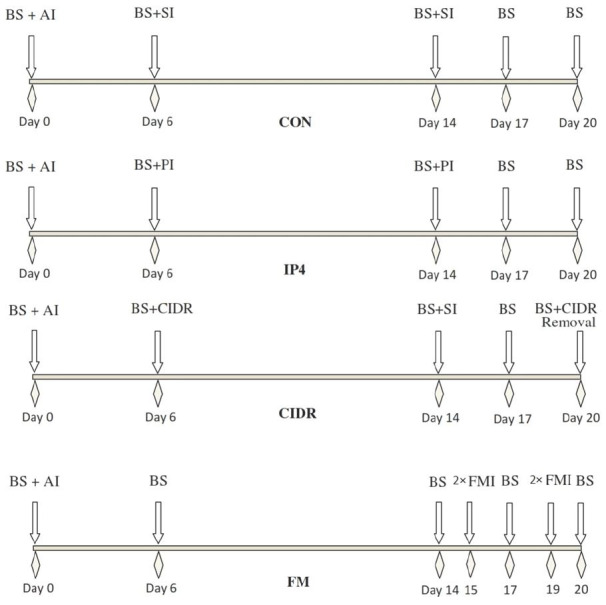
Illustration of experimental procedures and treatments during the study. Lactating dairy cows (n = 80) were randomly allocated to one of the following treatments groups: **CON** (control group: injection of saline on d 6 and 14 post AI); **IP4** (injection of 125 mg of progesterone im on d 6 and 14 post AI); **CIDR** (insertion of a controlled internal drug-release device containing 1.38g of P4 from d 6 to 20 post AI) or **FM** (injection of 0.625 g of Flunixin Meglumine im twice daily on d 15 and 19 post AI). AI (Artificial Insemination); BS (Blood Sampling); SI (Saline Injection); PI (Progesterone Injection); FM (Flunixin Meglumine).

Illustration of experimental procedures and treatments during the study are shown in Figure 1 .

Data were analyzed using SAS software (SAS 9.3; 2003; SAS Institute Inc., Cary, NC, USA). A probability of 0.05 or less was considered statistically significant, and a probability between 0.051 and 0.1 was considered a tendency.

Serum P_4_ data was analyzed with the MIXED procedure of SAS using the following model:

$$Y_{ijk}=\mu +C_{i}+T_{j}+D_{k}+TD_{jk}+\varepsilon_{ij}$$

Where $Y_{ij}$ is the dependent variable, μ is the overall mean, $C_{i}$ is the effect of cow *i*, $T_{j}$ is the effect of treatment *j*_,_ $D_{k}$ is the effect of sampling day *k*, $TD_{jk}$ is the interaction between treatment *j* and sampling day *k* and $\varepsilon_{ij}$ is the residual error. Two orthogonal contrasts were planned *a priori* to determine the differences between treatment groups (Contrast 1; FM *vs.* IP4 and CIDR and Contrast 2; IP4 *vs.* CIDR). Pregnancy per AI (P/AI) was compared among all experimental groups by using logistic procedure of SAS.

## Results

**Figure 2:**
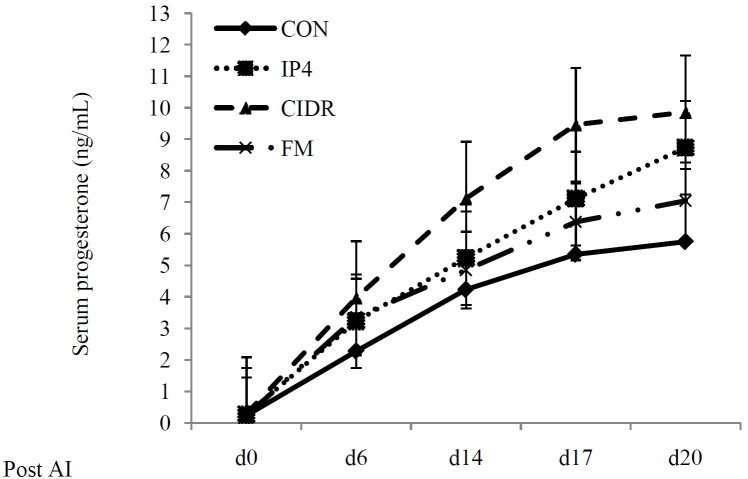
Serum progesterone (P_4_) concentrations (ng/mL) in all lactating dairy cows during the study. **CON** (♦): received saline solution im on d 6 and 14 post AI; IP4 (■): received 125 mg of P_4_ im on d 6 and 14 post AI; **CIDR** (▲): given a controlled internal drug-release device containing 1.38 g of P_4_ from d 6 to 20 post AI; **FM** (×): received 0.625 g of Flunixin Meglumine (FM), a non-steroidal anti-inflammatory drug, im twice daily on d 15 and 19 post AI.

The serum P4 concentrations in all cows during the entire study are presented and displayed in Table 1 and Figure 2 . Serum P_4_ concentrations at d 0 and 6 post AI did not differ (P > 0.05) among treatment groups. However, cows in the IP4, CIDR and FM groups tended to have greater (P = 0.06) serum P_ 4_ concentration at d 14 post AI and had greater (P < 0.01) serum P_4_ concentration at d 17 and 20 post AI compared to cows in the CON group. In addition, cows given a CIDR had greater (P < 0.01) concentrations of P_4_ in serum at d 17 and 20 post AI than IP4- or FM-treated animals. There was no significant difference for serum P_4_ concentration between IP4 and FM during the entire study.

**Table 1:** Serum progesterone concentrations (ng/mL) in lactating dairy cows treated with saline (CON), progesterone (IP4), CIDR, or flunixin meglumine (FM).

Treatments 1						Contrasts 2		
Sampling time points	CON	IP4	CIDR	FM		P	C1	C2
**d 0** **3**							
Non-Pregnant	0.221 ± 0.08	0.238 ± 0.07	0.263 ± 0.01	0.213 ± 0.07		0.93	0.71	0.80
Pregnant	0.245 ± 0.09	0.273 ± 0.09	0.297 ± 0.06	0.249 ± 0.05		0.75	0.67	0.73
Overall	0.234 ± 0.05	0.261 ± 0.04	0.285 ± 0.08	0.230 ± 0.09		0.81	0.74	0.84
**d 6**								
Non-Pregnant	2.11 ± 0.23	3.09 ± 0.43	3.78 ± 0.30	3.19 ± 0.31		0.36	0.27	0.23
Pregnant	2.40 ± 0.12	3.40 ± 0.18	4.12 ± 0.22	3.56 ± 0.23		0.67	0.56	0.17
Overall	2.28 ± 0.16	3.23 ± 0.14	3.97 ± 0.20	3.36 ± 0.15		0.42	0.38	0.11
**d 14**								
Non-Pregnant	3.91 ± 0.95	5.01 ± 1.09	5.29 ± 0.83	4.18 ± 0.79		0.12	0.23	0.13
Pregnant	4.42 ± 0.78	5.41 ± 0.96	8.23 ± 75	5.12 ± 0.74		0.05	0.06	0.03
Overall	4.23 ± 0.56	5.23 ± 0.75	7.12 ± 0.47	4.85 ± 0.86		0.06	0.08	0.09
**d 17**								
Non-Pregnant	3.23 ± 1.09	4.49 ± 0.45	5.37 ± 0.62	3.98 ± 0.57		0.34	0.18	0.21
Pregnant	6.42 ± 95	8.78 ± 0.73	10.19 ± 0.48	8.23 ± 0.97		<0.01	0.02	<0.01
Overall	5.35 ± 0.48	7.12 ± 0.87	9.46 ± 0.92	6.38 ± 0.57		<0.01	<0.01	<0.01
**d 20**								
Non-Pregnant	2.34 ± 0.35	4.98 ± 0.67	5.19 ± 0.96	4.79 ± 0.65		0.24	0.36	0.42
Pregnant	7.78 ± 0.95	11.87 ± 0.86	13.23 ± 1.25	9.21 ± 1.08		<0.01	<0.01	<0.01
Overall	5.75 ± 0.56	8.73 ± 0.67	9.86 ± 0.86	7.05 ± 0.71		<0.01	<0.01	0.02

1 CON: injection of saline on d 6 and 14 post AI; IP4: injection of 125 mg of P4 im on d 6 and 14 post AI; CIDR: administration of a controlled internal drug-release insert containing 1.38 g of P4 from d 6 to 20 post AI; FM: 1.25 g of Flunixin Meglumine, a non-steroidal anti-inflammatory drug, im twice daily on d 15 and 19 post AI.

2 Orthogonal contrasts; contrast 1 (C1), FM vs. IP4 and CIDR; and contrast 2 (C2), IP4 vs. CIDR.

3 day 0 = day of AI.

**Figure 3:**
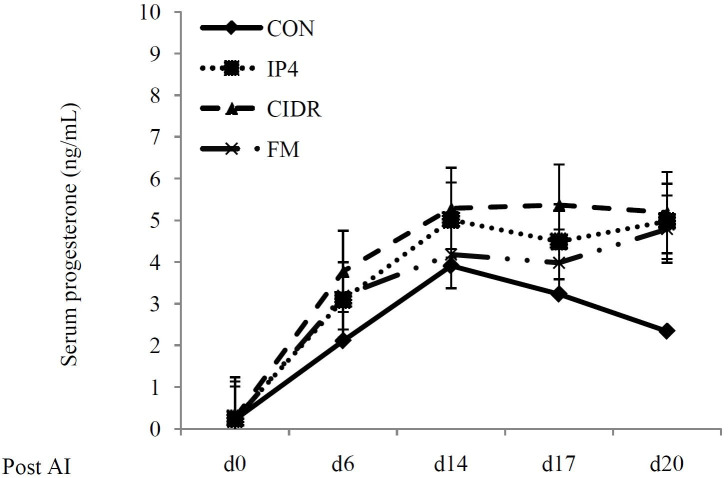
Serum progesterone (P_4_) concentrations (ng/mL) in non-pregnant lactating dairy cows during the study. **CON** (♦): received saline solution im on d 6 and 14 post AI; IP4 (■): received 125 mg of P_4_ im on d 6 and 14 post AI; **CIDR** (▲): given a controlled internal drug-release device containing 1.38 g of P_4_ from d 6 to 20 post AI; **FM** (×): received 0.625 g of Flunixin Meglumine (FM), a non-steroidal anti-inflammatory drug, im twice daily on d 15 and 19 post AI.

**Figure 4:**
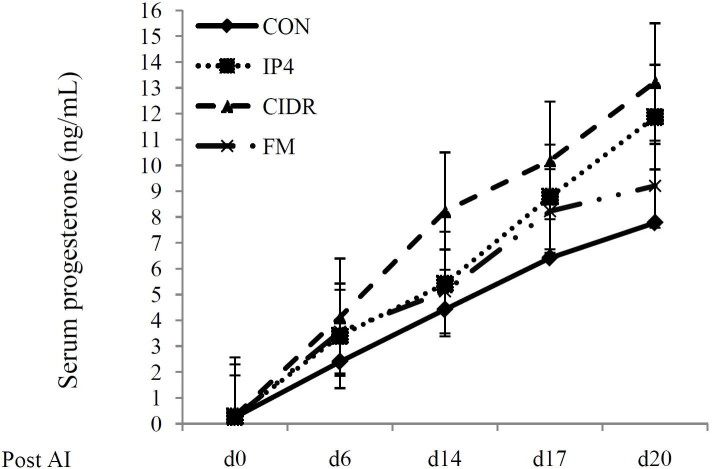
Serum progesterone (P_4_) concentrations (ng/mL) in pregnant lactating dairy cows during the study. **CON** (♦): received saline solution im on d 6 and 14 post AI; IP4 (■): received 125 mg of P_4_ im on d 6 and 14 post AI; **CIDR** (▲): given a controlled internal drug-release device containing 1.38 g of P_4_ from d 6 to 20 post AI; **FM** (×): received 0.625 g of Flunixin Meglumine (FM), a non-steroidal anti-inflammatory drug, im twice daily on d 15 and 19 post AI.

We also compared the serum P_4_ concentrations in pregnant ( Figure 3 ) and non-pregnant ( Figure 4 ) cows among CON, IP4, CIDR and FM groups. Serum P_4_ concentrations on d 0 and 6 post AI did not differ (P > 0.05) among treatment groups in pregnant and non-pregnant cows. Similarly, serum P_4_ concentrations on d 14, 17 and 20 post AI were not significantly different (P > 0.05) among treatment groups in non-pregnant cows. However, in pregnant cows, serum P_4_ concentrations on d 14 post AI were greatest (P < 0.05) in CIDR group compared to CON, IP4 or FM. Moreover, pregnant cows in IP4 and FM groups had greater (P < 0.05) serum P _4_ concentrations than pregnant cows in CON group. However, P_4_ concentrations did not differ between IP4 and FM groups (P < 0.05). On d 17 post AI, serum P_4_ concentrations were significantly greater in CIDR group (P < 0.01) compared to CON, IP4 or FM. Interestingly P_4_ concentrations did not differ between CON and FM groups. However, pregnant cows in the IP4 group had greater (P < 0.01) P_4_ concentrations than pregnant cows in the CON group. Cows in the CIDR group also had the greatest serum P4 concentrations on d 20 post AI (P < 0.01). In addition, cows in FM and IP4 groups had greater P_4_ concentrations than cows in CON group (P < 0.01). However, serum P_4_ concentrations did not differ between IP4 and FM groups (P > 0.05).

The P/AI for CON, IP4, CIDR and FM groups were 25% (5/20), 40% (8/20), 55% (11/20) and 35% (7/20), respectively. There was a significant difference for P/AI between CIDR and CON groups (P = 0.03). However, P/AI did not differ between IP4 and FM groups (P = 0.58) or when both groups were compared with CON (P = 0.17).

## Discussion

Progesterone is essential to successful establishment of pregnancy in cattle and inadequate circulating P_4_ is one of the main reasons of low fertility in lactating dairy cows ( Inskeep, 2004 ; Dirandeh *et al*., 2018 ). The present study evaluated the effect of post AI administration of exogenous P_4_ or a PGF_2_α synthesis inhibitor agent on serum P_4_ concentrations in lactating dairy cows. There were no significant differences for serum P_4_ concentrations among treatment groups on d 0 and 6 post AI in pregnant and non-pregnant cows. This was expected as treatments were initiated on d 6 or after d 6 post AI. However, serum P_4_ concentrations were affected by treatments on d 14, 17 and 20 post AI. Cows given exogenous P_4_, in particular those receiving a CIDR insert, tended to have greater P_4_ concentrations on d 14 post AI compared to those receiving FM or control group. On d 17 and 20, the average serum P_4_ concentration remained greater in cows given a CIDR insert but it was not statistically different between cows in IP_4_ and FM groups. Control cows had lower serum P_ 4_ concentrations compared to all three treatment groups. The differences in serum P_ 4_ concentrations on d 20 post AI between treatment groups and control might have also been associated to the pregnancy status of the cows, rather than merely to the treatment per se, as numerically more cows were pregnant in all three treatment groups compared to control.

The pharmacokinetics of P_4_ following CIDR insertion has been previously evaluated in cattle ( Martinez *et al.*, 2007 ; Mariano *et al.*, 2010 ). In a study, ovariectomized beef cows received a previously used CIDR insert for 7 d. Plasma P_4_ concentrations increased to ∼2 ng/mL by 6 h after CIDR insertion. Thereafter, plasma P_4_ concentrations decreased by 84 h after CIDR insertion and remained relatively constant. By 12 h after CIDR removal on day 7, P_4_ concentrations declined to <0.2 ng/mL ( Martinez *et al.*, 2007 ). In another study using lactating dairy cows without a functional CL, serum P_4_ peaked within 3 h after CIDR insertion followed by a progressive decrease over the next 2 d until reaching sustained concentrations over the remaining 6 d of CIDR treatment. The range of sustained P_4_ serum concentrations varied between 2.25 and 4.09 ng/mL ( Mariano *et al.*, 2010 ). In the present study, serum P_4_ concentrations increased from ∼4 to ∼9 ng/mL by 11 d after CIDR insertion and remained relatively constant until CIDR removal (d 20 post AI) in CIDR-treated cows.

Neither the pharmacokinetics of P_4_ following intramuscular injections nor their effect on serum P_4_ concentrations and PR/AI has been reported in lactating dairy cows. However, Turino *et al.* (2010) examined the pharmacokinetics of P_4_ in lactating dairy cows given an intravenous injection of 100 mg P_4_. Plasma P_4_ reached a concentration of 140 ng/mL 1 min after treatment and dropped to baseline concentrations within 2 h after treatment ( Turino *et al.*, 2010 ). In our study, serum P_4_ concentrations increased from ∼3 (day 6 post AI) to ∼5 ng/mL (day 14 post AI) and from ∼5 (day 14 post AI) to∼ 9 ng/mL (day 20 post AI) in cows given P4 im.

Multiple factors can affect the very complex process of pregnancy maintenance during the time of recognition of pregnancy. During implantation, appropriate antiluteolytic signals i.e. interferon-tau produced by conceptus is vital to prevent endometrial PGF_2_α secretion ( Poyser, 1995 ; Dirandeh *et al*., 2015 ). Conversely, during maternal recognition of pregnancy, interferon-tau increases endometrial prostaglandin E_2_ (PGE_2_), which is considered a potent luteoprotective factor ( Arosh *et al*., 2016 ). Interestingly, endometrial PGE_2_ induces additional PGE_2_ biosynthesis from CL which counteracts the luteolytic effect of PGF_2_α during maternal recognition of pregnancy or at the time of establishment of pregnancy ( Romero *et al*., 2015 ). Agents that prevent the synthesis of PGF_2_α such as Flunixin Meglumine or Meloxicam are licensed to be used in cattle and can potentially extend the CL life span. These agents are classified as non-steroidal anti-inflammatory drugs (NSAIDs) which may act on different prostaglandin pathways (i.e. COX-1 and COX-2). Flunixin Meglumine is both a COX-1 and COX-2 inhibitor but is more selective for COX-1; it has an elimination half-life of 3 to 8 h ( Odensvik, 1995 ). In a study, supplementation with 1 g of FM during the first 6 d postpartum decreased the release of PGF_2_α as mirrored by decreased plasma concentrations of 13, 14-dihydro-15-keto-PGF _2_α in brown Swiss cows ( Guilbault *et al*., 1987 ).

The current study also evaluated the effect of post AI administration of exogenous P_4_ or a PGF_2_α synthesis inhibitor agent on P/AI in lactating dairy cows. It has been shown that low circulating P_4_ concentrations or a delay in the rise of P_ 4_ during the early post ovulatory phase is associated with reduced conceptus development and fertility in cattle ( Mann and Lamming, 1999 ; Masoumi *et al*., 2012 ; Masoumi *et al*., 2017 ). Several studies have evaluated the effect of post AI supplementation with exogenous P_ 4_ during metaestrus or early diestrus on fertility of lactating dairy cows. Administration of a previously used CIDR, which originally contained 1.9 g of P_4_, from day 3.5 to day 10 post AI resulted in improved P/AI of dairy cows ( Larson *et al*., 2007 ). However, other researchers ( Arndt *et al*., 2009 ; Colazo *et al*., 2013 ) did not observe any improvement in P/AI in lactating cows treated with a CIDR containing 1.38 g of P_4_ or a PRID containing 1.55 g P_4_, from d 4 to 18 or 4.5 to 11.5 post AI, respectively.

A research group from Spain has investigated the effect of P_4_ supplementation during late diestrus or at the time of maternal recognition of pregnancy on reproductive performance of high producing dairy cows. Circulating concentrations of P_4_ and fertility did not increase in dairy cows given an insert containing 1.55 g of P_4_ from d 5 to 19 post AI ( Garcia-Ispierto and López-Gatius, 2012 ). In a more recent study, Garcia-Ispierto *et al.* (2016) determined fertility response to P_4_ supplementation from day 15 to 17 post-AI in high-producing dairy cows. Cows with no history of early postpartum reproductive disorders (i.e. retained placenta) that were supplemented with P_4_ during maternal recognition of pregnancy were 1.6 times more likely to become pregnant than the control (no P_4_ treatment) herd mates.

In the present study, P/AI was enhanced in cows given a CIDR insert compared to control cows. Albeit, no statistically significant, P/AI in IP4 cows was 14% greater than in control cows. It is noteworthy to mention that the effect of P_4_ on fertility was not the primary objective of this study, which was under powered to detect a 14% difference in P/AI among treatments. However, our results are in agreement with others and support the notion that P_4_ supplementation during early and late diestrus has a positive effect on P/AI in lactating dairy cows. In addition, numerically more FM cows (35%) become pregnant compared to control cows (25%), but the difference in P/AI was not statistically significant. Current research has not investigated the effect of administration of FM on P/AI in lactating dairy cows, but several reports have been published regarding the effect of FM on P/AI in dairy heifers. In agreement with our results, dairy heifers (n = 2325) treated twice with 400 mg of FM im at d 15 and 16 post AI had similar P/AI compared to untreated heifers (59.4 vs 59.5%) ( Rabaglino *et al*., 2010 ). However, Guzeloglu *et al.* (2007) reported that the administration of 1.1 mg/kg FM 12 h apart at 15.5 and 16 d after timed-artificial insemination (TAI) improved P/AI by 23% in dairy heifers (69.2 vs 46.2%). Also, FM has been administered to prevent early regression of the CL and increase pregnancy rate in recipient cattle immediately before embryo transfer ( Schrick *et al*., 2001 ; Aguiar *et al*., 2013 ; Kasimanickam *et al*., 2018 ). Amiridis *et al.* (2009) assessed the effectiveness of three different NASIDs including ketoprofen, Meloxicam and FM on the length of estrous cycle and showed that Meloxicam was the most potent among the three NAIDs. In addition, Aguiar *et al.* (2013) reported that Meloxicam had a positive effect on the overall pregnancy rate of embryo recipient heifers. However, pregnancy rate was not affected by administration of Meloxicam recipient heifers classified as Grade I (easy passing catheter) but Meloxicam increased the pregnancy rate of heifers classified as Grade II (difficult passing catheter). Therefore, the effect of the administration of FM or Meloxicam on P/AI in lactating dairy cows warrants further investigation.

## Conclusions

In summary, treatment with exogenous P_4_ (i.e. CIDR insertion from d 6 to 20 post AI or P_4_ injection on d 6 and 14 post AI) or Flunixin Meglumine (PGF2α synthesis inhibitor; twice daily on d 15 and 19 post AI) increased serum P_4_ concentrations compared to treatment with saline solution (control). However, results suggest that only CIDR administration would improve P/AI in lactating dairy cows.
